# COVID-19 Patient Management in Outpatient Setting: A Population-Based Study from Southern Italy

**DOI:** 10.3390/jcm11010051

**Published:** 2021-12-23

**Authors:** Salvatore Crisafulli, Valentina Ientile, Luca L’Abbate, Andrea Fontana, Claudio Linguiti, Sonia Manna, Mariangela Mercaldo, Claudia Pagliaro, Michele Vezzaro, Katia Santacà, Riccardo Lora, Ugo Moretti, Chiara Reno, Maria Pia Fantini, Salvatore Corrao, Donato Barbato, Michele Tari, Gianluca Trifirò, the ITA-COVID: COV-OUT Group

**Affiliations:** 1 Department of Medicine, University of Verona, 37134 Verona, Italy; salvatore.crisafulli@univr.it; 2 Department of Biomedical and Dental Sciences and Morphofunctional Imaging, University of Messina, 98125 Messina, Italy; vientile@unime.it; 3 Department of Diagnostics and Public Health, University of Verona, 37134 Verona, Italy; luca.labbate@univr.it (L.L.); michele.vezzaro@univr.it (M.V.); katia.santaca@univr.it (K.S.); riccardo.lora@univr.it (R.L.); ugo.moretti@univr.it (U.M.); 4 Unit of Biostatistics, Fondazione IRCCS Casa Sollievo della Sofferenza, 71013 San Giovanni Rotondo, Italy; a.fontana@operapadrepio.it; 5 Caserta Local Health Unit, 81100 Caserta, Italy; claudio.linguiti@aslcaserta.it (C.L.); sonia.manna@aslcaserta.it (S.M.); mariangela.mercaldo@aslcaserta.it (M.M.); claudia.pagliaro@aslcaserta.it (C.P.); michele.tari@aslcaserta.it (M.T.); 6 Department of Biomedical and Neuromotor Sciences, Alma Mater Studiorum, University of Bologna, 40126 Bologna, Italy; chiara.reno@studio.unibo.it (C.R.); mariapia.fantini@unibo.it (M.P.F.); 7 Department of Health Promotion Sciences, Maternal and Infant Care, Internal Medicine and Medical Specialties (PROMISE), University of Palermo, 90127 Palermo, Italy; salvatore.corrao@unipa.it; 8 Department of Internal Medicine, National Relevance and High Specialization Hospital Trust ARNAS Civico, Di Cristina, Benfratelli, 90127 Palermo, Italy; 9 IQVIA, 20124 Milan, Italy; donato.barbato@iqvia.com

**Keywords:** COVID-19, outpatients, Italy

## Abstract

Evidence on treatments for early-stage COVID-19 in outpatient setting is sparse. We explored the pattern of use of drugs prescribed for COVID-19 outpatients’ management in Southern Italy in the period February 2020–January 2021. This population-based cohort study was conducted using COVID-19 surveillance registry from Caserta Local Health Unit, which was linked to claims databases from the same catchment area. The date of SARS-CoV-2 infection diagnosis was the index date (ID). We evaluated demographic and clinical characteristics of the study drug users and the pattern of use of drugs prescribed for outpatient COVID-19 management. Overall, 40,030 patients were included in the analyses, with a median (IQR) age of 44 (27–58) years. More than half of the included patients were asymptomatic at the ID. Overall, during the study period, 720 (1.8%) patients died due to COVID-19. Azithromycin and glucocorticoids were the most frequently prescribed drugs, while oxygen was the less frequently prescribed therapy. The cumulative rate of recovery from COVID-19 was 84.2% at 30 days from ID and it was lower among older patients. In this study we documented that the drug prescribing patterns for COVID-19 treatment in an outpatient setting from Southern Italy was not supported from current evidence on beneficial therapies for early treatment of COVID-19, thus highlighting the need to implement strategies for improving appropriate drug prescribing in general practice.

## 1. Introduction

The severe acute respiratory syndrome coronavirus 2 (SARS-CoV-2) is a novel coronavirus causing the coronavirus disease (COVID-19). A considerable body of evidence on COVID-19 management in the hospital setting has been cumulated, while much less evidence on beneficial treatments for COVID-19 in an outpatient setting is available. To date, only anti-spike monoclonal antibodies received emergency use authorization by some regulatory agencies [1,2,3] for early treatment of mild-to-moderate COVID-19 outpatients, who are at high risk of progressing to severe disease. Moreover, as potential early COVID-19 outpatient treatment, European Medicine Agency (EMA) is currently evaluating the oral direct-acting antiviral molnupiravir, which was proven to be effective at reducing SARS-CoV-2 viral RNA in a phase 2 clinical trial [4]. In a real-world setting, drugs such as non-steroidal anti-inflammatory drugs (NSAIDs), glucocorticoids, azithromycin, and heparins have been frequently used for COVID-19 outpatient management, under the hypothesis that these drugs may somehow block or delay COVID-19 progression [5,6]. To date, no population-based studies on the pattern of drugs used for COVID-19 management in an Italian outpatient setting have been published.

A small recent Italian retrospective cohort study including 90 patients treated at home by general practitioners suggested that early NSAID treatment in COVID-19 outpatients may prevent disease progression [7]. However, this study was not able to demonstrate any effect of these drugs on time to complete remission.

Antibiotics are recommended for COVID-19 treatment only in case of persistent symptoms leading to suspicion of bacterial superinfections/co-infections [8]. Due to its supposed antiviral and immunomodulatory effects, azithromycin has been however extensively prescribed in COVID-19 patients, [9], despite a large body of evidence that has documented no benefits of this antibiotic, beyond its antibacterial effect [9].

Treatment with low-molecular weight heparins is recommended for the prophylaxis of thromboembolic events in COVID-19 hospitalized adult patients with acute respiratory infection and reduced mobility, while it is not recommended for non-hospitalized/non-bedridden patients [8,10].

Based on meta-analyses of randomized controlled trials (RCTs) [11,12], corticosteroids are strongly recommended for COVID-19 patients requiring oxygen support. Conversely, early treatment with these drugs may be detrimental as high-dose or prolonged glucocorticoid therapy has been associated with increased risk of SARS-CoV-2 infection and negative COVID-19-related outcomes [13,14].

This population-based study aimed to explore the pattern of drug use for COVID-19 management in an outpatient setting from a large province of Southern Italy in the period 21 February 2020–31 January 2021.

## 2. Materials and Methods

### 2.1. Data Source

A retrospective population-based cohort study was carried out using COVID-19 surveillance registry from Caserta Local Health Unit (LHU) that was linked to claims databases from the same catchment area. In Italy, residents have access to universal healthcare services that are provided by National Health System (NHS) and are traced through administrative claims databases, which are widely used for clinical research. We used in this study claims data providing information on hospital admissions, copayment exemptions, and pharmacy claims. Diagnoses were coded using the International Classification of Diseases, 9th Revision, Clinical Modification (ICD-9-CM), while drugs were recorded using the Anatomical Therapeutic Chemical (ATC) classification system. Claims databases were linked to local COVID-19 surveillance registry through unique fully-anonymized patient identifiers. This registry was used to identify patients testing positive for SARS-CoV-2 RNA by polymerase chain reaction (PCR) on nasopharyngeal/throat swabs and related outcomes.

Some of the study drugs may not be captured by claims databases as out-of-pocket purchases. To assess the proportion of drug units, which were not purchased through NHS, from Farma360 [15], pharmacy sales data in the period February 2020–February 2021 was extracted.

### 2.2. Study Population

The study population consisted of all Caserta LHU residents with at least one SARS-CoV-2 infection diagnosis, as reported in the COVID-19 registry during the period 21 February 2020–31 January 2021. Given that COVID-19 surveillance registry and pharmacy claims were respectively updated till 2 April 2021 and 28 February 2021, the end of the enrolment period was set to 31 January 2021, with the aim to have at least one month of follow-up for every enrolled patient. The date of the first laboratory-confirmed SARS-CoV-2 infection diagnosis during the enrolment period was considered as the index date (ID).

### 2.3. Exposure Definition

SARS-CoV-2 positive patients were classified as users of any of the study drug if receiving ≥1 pharmacy claim during the period ranging from 10 days prior to ID to the end of the observation period (date of recovery or death, or 28 February 2021) in outpatient setting. As exposure of interest, the following non-mutually exclusive categories were considered: (i) azithromycin users; (ii) glucocorticoid users; (iii) heparin users; (iv) oxygen therapy recipients; (v) other antibiotic users; (vi) vitamin D and analogues users (Supplementary Table S1). NSAIDs are frequently purchased out of pocket and, as such, these drugs may not be accurately traced through claims databases. As a consequence, NSAIDs were not included in the final analysis. Furthermore, we did not include hydroxychloroquine as it was extensively used only during the first wave of the pandemic.

### 2.4. Outcome Definition

On the basis of the information collected into the COVID-19 surveillance registry, patients were classified as: (i) recovered, if they had only a recovery date after ID or both a recovery date and a death date, but the latter was recorded ≥15 days after the recovery date; (ii) deceased, if they had only a date of death or both a recovery date and a death date, but the latter was recorded within 15 days after the recovery date; (iii) not yet recovered, if they had neither the date of recovery nor the date of death, until the end of the observation period.

### 2.5. Data Analysis

The monthly frequency distribution of SARS-CoV-2 infection diagnoses during the study period was evaluated. Demographic and clinical characteristics of the patients with confirmed SARS-CoV-2 infection during the observation period were analyzed and stratified by study drug use (evaluated at the ID) and clinical outcome, separately. Study variables included patients’ demographics, comorbidities, prior use of drugs and SARS-CoV-2 infection-related symptoms and were reported as median along with interquartile range (IQR) and frequency for continuous and categorical variables, respectively. Comorbidities were identified through search of specific coding algorithms (Supplementary Table S2), any time prior to ID, in claims databases, while prior use of drugs was searched within 12 months prior to ID (Supplementary Table S3). SARS-CoV-2 infection-related symptoms at the time of confirmed diagnosis were evaluated using COVID-19 registry. For each exposure of interest, the proportion of incident users (i.e., subjects without any prescription of the same study drug within the three months prior to the date of the first study drug dispensing) was evaluated.

The follow-up time was defined as: (i) for recovered patients: the time between ID and the recovery date; (ii) for deceased patients: the time between ID and death date; (iii) for not yet recovered patients: the time between ID and 21 days after ID unless COVID-19-related hospitalization occurred before. The maximum allowed follow-up time of COVID-19 patients was equal to six months.

We measured the monthly frequency distribution of individual drug/drug class use as well as the frequency of co-prescriptions of different study drugs. We carried out an age and sex adjusted competing risk analysis considering recovery or death as the only causes of failure.

For each incident user of the study drugs, the time elapsed between ID and the date of the first drug dispensing was computed and aggregated data were stratified by individual study drug/drug class and symptom severity at ID. For study drug users with the first pharmacy claim recorded within 10 days prior the ID, the observation period was defined as the negative difference between the date of the pharmacy claim and the ID. The observation period was defined as follows: (i) for recovered patients, from ID to 15 days after the date of recovery; (ii) for deceased patients, from ID to the date of death; (iii) for patients who died within 15 days from the date of recovery, from ID to the date of death; (iv) for patients with only the date of COVID-19-related hospitalization, from ID to the date of hospitalization; (v) for not yet recovered patients, from ID to 21 days after ID.

The monthly proportion of drug packages that were out-of-pocket purchased was calculated as the number of package purchases without NHS reimbursement divided by all package purchases for each calendar month.

Moreover, time to recovery from SARS-CoV-2 infection from ID, stratified by age and sex, was evaluated, considering recovery or death as the only causes of failure. This analysis was also carried out adjusting by study drug use and the most commonly reported comorbidities.

Statistical analyses were performed using SAS 9.4 (SAS Institute, Cary, NC, USA) and R Foundation for Statistical Computing, Vienna, Austria (version 4.04).

## 3. Results

**Figure 1:**
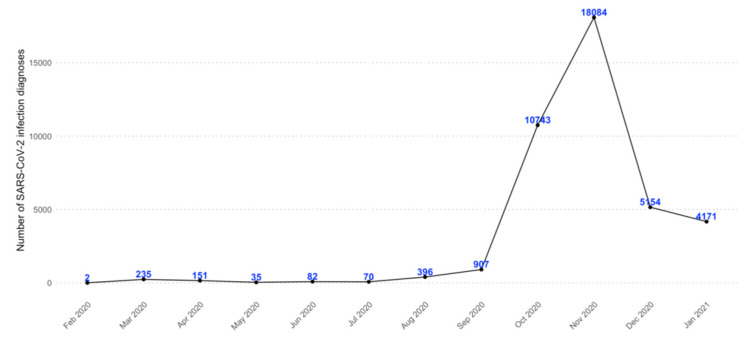
Monthly frequency of SARS-CoV-2 infection confirmed diagnoses in the period 21 February 2020–31 January 2021, in Caserta Local Health Unit.

During the period 21 February 2020–31 January 2021, overall 40,030 COVID-19 patients from Caserta LHU were identified. Most of these patients (N = 33,981; 84.9%) were newly diagnosed with SARS-CoV-2 infection in the period October–December 2020 (Figure 1).

Overall, the male/female ratio of COVID-19 patients was equal to 1.0, even if deceased patients were mostly males (N = 417; 57.9%); the median age of COVID-19 patients was 44 (IQR: 27–58) years. More than half of these patients were asymptomatic at the time of diagnosis (Supplementary Table S4).

During the observation period, azithromycin was the most frequently prescribed study drug (N = 16,843; 42.1%) in COVID-19 outpatients, followed by glucocorticoids (N = 14,412; 36.0%), vitamin D (N = 9486; 23.7%), and other antibiotics (N = 8360; 20.9%). Among glucocorticoids, the most commonly used was prednisone (N = 12,324; 85.5%), followed by betamethasone (N = 2029; 14.1%) and dexamethasone (N = 1584; 11.0%). More than 90% of study drug users were overall incident, with a slightly lower extent for vitamin D and other antibiotics users (82.8% and 76.2%, respectively).

As compared to other treatment groups, patients receiving oxygen had higher median age (66 (IQR: 55–77) years) and overall worse clinical conditions. Among all treatment groups, hypertension and diabetes were the most commonly reported comorbidities, while drugs for acid-related disorders and lipid-lowering drugs were the most frequently co-prescribed drugs. More than 30% of patients in each treatment group were asymptomatic at ID (Table 1).

**Table 1:** Demographic and clinical characteristics of COVID-19 patients from Caserta Local Health Unit, stratified by the study drug, assessed at the time of SARS-CoV-2 infection diagnosis in the period 21 February 2020–31 January 2021.

	Glucocorticoids N = 14,412 (%)	Azithromycin N = 16,843 (%)	Other Antibiotics N = 8360 (%)	Heparins N = 6322 (%)	Vitamin D N = 9486 (%)	Oxygen N = 2664 (%)
**% on Total Number of Patients with Confirmed SARS-CoV-2 Infection Diagnosis**						
**Incident users ^a^**	13,258 (91.9)	16,145 (95.9)	6368 (76.2)	5995 (94.8)	7862 (82.8)	2568 (96.4)
**Sex**						
Males	7083 (49.1)	8287 (49.2)	4191 (50.1)	3196 (50.6)	3876 (40.9)	1533 (57.5)
Females	7329 (50.9)	8556 (50.8)	4169 (49.9)	3126 (49.4)	5610 (59.1)	1131 (42.5)
**Median age (IQR) (years)**	50 (37–63)	49 (34–61)	56 (43–69)	59 (48–71)	52 (38–64)	66 (55–77)
**Age groups (years)**						
<18	462 (3.2)	878 (5.2)	259 (3.1)	24 (0.4)	313 (3.3)	4 (0.2)
18–44	5077 (35.2)	6218 (36.9)	2081 (24.9)	1216 (19.2)	3042 (32.1)	261 (9.8)
45–64	5790 (40.2)	6542 (38.8)	3436 (41.1)	2762 (43.7)	3906 (41.2)	1033 (38.8)
65–80	2245 (15.6)	2416 (14.3)	1830 (21.9)	1608 (25.4)	1656 (17.5)	845 (31.7)
>80	838 (5.8)	789 (4.7)	754 (9.0)	712 (11.3)	569 (6.0)	521 (19.6)
**Comorbidities ^b^**						
Hypertension	6754 (46.9)	7348 (43.6)	4875 (58.3)	4152 (65.7)	4764 (50.2)	2022 (75.9)
Ischemic cardiopathy	773 (5.4)	840 (5.0)	713 (8.5)	587 (9.3)	537 (5.7)	343 (12.9)
Atrial fibrillation	313 (2.2)	314 (1.9)	260 (3.1)	183 (2.9)	183 (1.9)	158 (5.9)
Heart failure	230 (1.6)	235 (1.4)	197 (2.4)	170 (2.7)	161 (1.7)	132 (5.0)
Cerebrovascular diseases	599 (4.2)	579 (3.4)	550 (6.6)	461 (7.3)	405 (4.3)	291 (10.9)
Diabetes mellitus	1608 (11.2)	2008 (11.9)	1544 (18.5)	1398 (22.1)	1353 (14.3)	737 (27.7)
Chronic kidney disease	239 (1.7)	239 (1.4)	219 (2.6)	197 (3.1)	165 (1.7)	130 (4.9)
Chronic pulmonary diseases	654 (4.5)	698 (4.1)	551 (6.6)	450 (7.1)	408 (4.3)	255 (9.6)
Hepatopathies	478 (3.3)	522 (3.1)	380 (4.5)	328 (5.2)	320 (3.4)	171 (6.4)
Neoplasms	1157 (8.0)	1261 (7.5)	861 (10.3)	763 (12.1)	845 (8.9)	377 (14.2)
**Prior use of drugs ^c^**						
Drugs for acid-related disorders	5647 (39.2)	5932 (35.2)	4293 (51.4)	3625 (57.3)	4269 (45.0)	1748 (65.6)
Lipid-lowering drugs	2609 (18.1)	2864 (17.0)	2094 (25.0)	1826 (28.9)	2017 (21.3)	920 (34.5)
Anti-platelet agents	1839 (12.8)	1945 (11.5)	1615 (19.3)	1437 (22.7)	1379 (14.5)	789 (29.6)
Anticoagulants (excl. heparins)	427 (3.0)	419 (2.5)	362 (4.3)	202 (3.2)	279 (2.9)	238 (8.9)
Class I and III antiarrhythmics	280 (1.9)	296 (1.8)	239 (2.9)	176 (2.8)	198 (2.1)	123 (4.6)
Anti HIV drugs	47 (0.3)	46 (0.3)	45 (0.5)	34 (0.5)	37 (0.4)	19 (0.7)
Anti-Parkinson drugs	162 (1.1)	156 (0.9)	161 (1.9)	145 (2.3)	111 (1.2)	95 (3.6)
Antiepileptics	668 (4.6)	720 (4.3)	522 (6.2)	435 (6.9)	467 (4.9)	226 (8.5)
Antipsychotics	346 (2.4)	351 (2.1)	293 (3.5)	239 (3.8)	239 (2.5)	133 (5.0)
Antidepressants	1111 (7.7)	1149 (6.8)	827 (9.9)	670 (10.6)	825 (8.7)	359 (13.5)
**Symptoms at the date of SARS-CoV-2 diagnosis**						
Asymptomatic	5975 (41.5)	7192 (42.7)	3347 (40.0)	2527 (40.0)	4006 (42.2)	869 (32.6)
Mild	4931 (34.2)	5796 (34.4)	2698 (32.3)	1893 (29.9)	3224 (34.0)	759 (28.5)
Moderate	2650 (18.4)	2956 (17.6)	1685 (20.2)	1399 (22.1)	1739 (18.3)	692 (26.0)
Serious	402 (2.8)	394 (2.3)	301 (3.6)	284 (4.5)	281 (3.0)	216 (8.1)
Missing values	454 (3.2)	505 (3.0)	329 (3.9)	219 (3.5)	236 (2.5)	128 (4.8)
**Patients with oximetry evaluation**	1592 (11.0)	1576 (9.4)	1157 (13.8)	1089 (17.2)	984 (10.4)	707 (26.5)
Median value at ID (IQR)	96 (94–98)	97 (95–98)	96 (94–98)	96 (93–97)	96 (94–98)	94 (91–96)

Note: Study drug use was evaluated in the period between 10 days prior to the SARS-CoV-2 infection diagnosis date (ID) and the end of the observation period; Abbreviations: IQR = interquartile range; SD = standard deviation; ^a^ subjects without any prescription of the same study drug in the three months preceding the date of the first study drug dispensing; ^b^ evaluated any time prior to the SARS-CoV-2 infection diagnosis date; ^c^ evaluated during the 12 months preceding the date of the first study drug. Overall monthly frequency of study drug use in COVID-19 patients increased substantially from September 2020 to January 2021, as compared to the previous months, with azithromycin and glucocorticoids being the most frequently prescribed drugs over time in outpatient setting (Figure 2).

**Figure 3:**
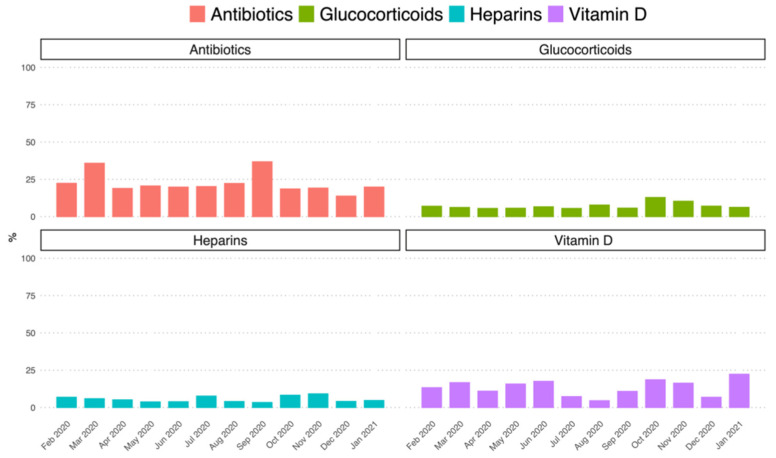
Frequency (%) of study drugs purchased privately in Caserta Local Health Unit on the total purchase, stratified for each calendar month in the period 1 February 2020–31 January 2021 and stratified by study drugs/drug classes. Note: Data on azithromycin private purchase was not available, therefore the private purchase of all antibiotics during the study period was evaluated. Oxygen was not included in this analysis because private purchase may be substantially underestimated due to the possibility of refilling oxygen cylinders.

During the study period, the mean proportion of purchased packages without NHS reimbursement was around 20% for antibiotics (including azithromycin) and 13.4%, 7.2%, and 5.5% for vitamin D, glucocorticoids, and heparins, respectively (Figure 3).

**Figure 4:**
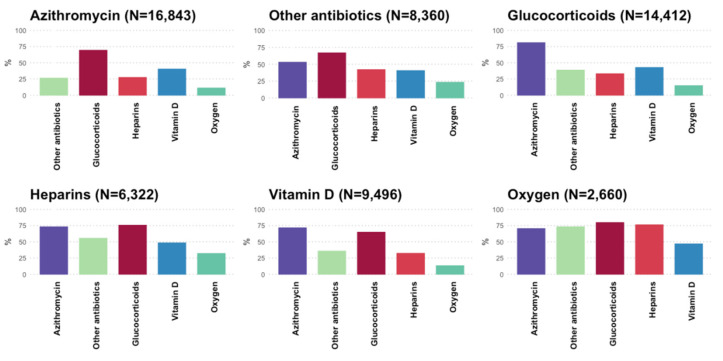
Frequency (%) of co-prescribed study drugs in Caserta Local Health Unit on the total number of each study drug users, in patients with a SARS-CoV-2 infection diagnosis that occurred in the period 21 February 2020–31 January 2021, stratified by active substance/drug class.

Overall, azithromycin was co-prescribed with other study drugs in ≥50% of COVID-19 patients, and up to 81% of those treated with glucocorticoids. More than one-quarter of azithromycin users also received other antibiotics for COVID-19 treatment (Figure 4).

**Figure 5:**
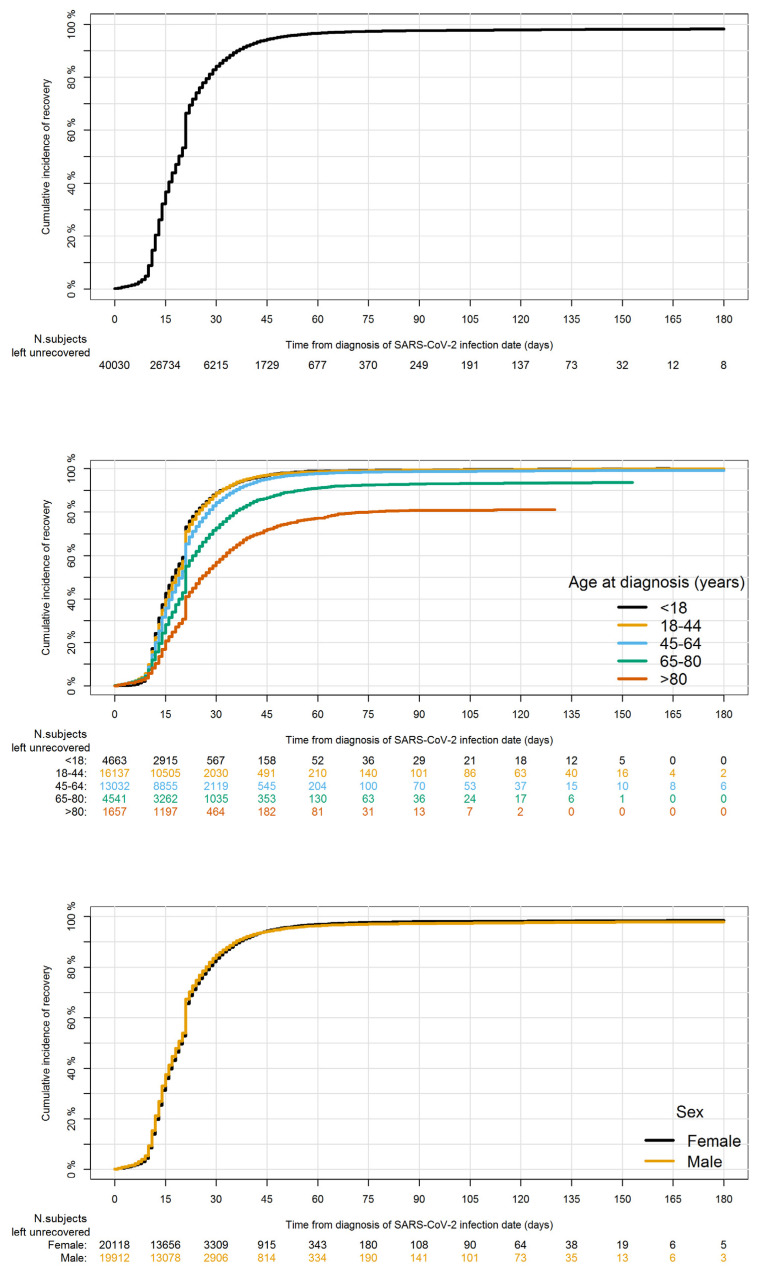
Cumulative recovery rate within 180 days from the first laboratory-confirmed SARS-CoV-2 infection diagnosis date in Caserta Local Health Unit in the period 21 February 2020–2 April 2021, in the overall population and stratified by age at diagnosis groups and sex.

Overall, 39,179 (97.9%) patients recovered from COVID-19 during the study period, while 720 (1.8%) patients died, and 131 (0.3%) were not yet recovered. The cumulative recovery rate was 84.2% at 30 days from ID, with a breakpoint at 21 days from ID and it was higher among younger patients (88.9% among patients with <18 years and 88.6% among patients in the age group 18-44) and lower among older patients (72.8% among patients in the age group 65–80 years and 56.9% among patients aged >80 years). No differences between males and females were observed (Figure 5).

The cumulative COVID-19-related mortality rate at 30 days from ID was 1.6% and it was higher among older patients (16.4% in patients that were 80 years old and more) and males than females (1.9% vs. 1.3%) (Supplementary Figure S1).

**Figure 6:**
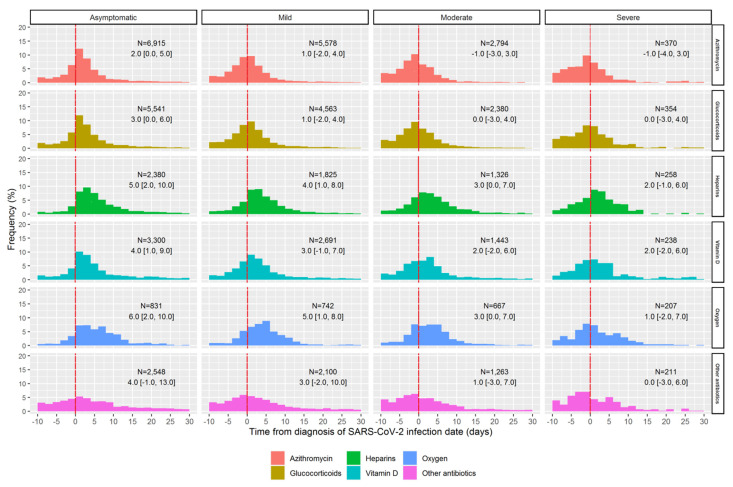
Distribution of the time elapsed between the date of the first laboratory-confirmed SARS-CoV-2 infection diagnosis and the date of the first pharmacy claim, stratified by study drugs/drug classes and symptoms at the SARS-CoV-2 diagnosis among incident drugs users. Note: The number of users (N) and the median (along with IQRs) of elapsed days are reported within each plot, respectively.

The median time that elapsed between ID and first dispensing date was 1 day for azithromycin, 2 days for glucocorticoids and other antibiotics, 3 days for vitamin D, and 4 days for oxygen and heparins. Around one-third of patients treated with glucocorticoids (N = 4544; 31.5%) or azithromycin (N = 5725; 34.0%) started the treatment within 10 days before SARS-CoV-2 infection diagnosis confirmation. By restricting the analysis only to incident users, in general, patients with mild-to-severe symptoms at ID started treatment earlier than asymptomatic patients, and many of them received the first drug dispensing before diagnostic confirmation of SARS-CoV-2 infection (Figure 6).

Overall, the median time elapsed between ID and the date of the first study drug dispensing was slightly longer in recovered patients than those deceased or not yet recovered (Supplementary Figure S2). The same analysis carried out adjusting by study drug use showed that patients who were asymptomatic at ID recovered faster than those with mild-to-severe symptoms at ID (Supplementary Figure S3). Furthermore, we did not observe significant differences in terms of time to recovery when stratifying for the six most commonly reported comorbidities (i.e., hypertension, ischemic cardiopathy, cerebrovascular diseases, diabetes mellitus, chronic pulmonary diseases, neoplasms) (Supplementary Figure S4).

## 4. Discussion

To our knowledge, this is the first population-based study from Italy exploring COVID-19 pharmacological management in an outpatient setting in the period preceding the anti-spike monoclonal antibodies marketing. Overall, findings from this study were in line with the results of the monitoring of drug use carried out by AIFA, using aggregated data [16].

A massive use of azithromycin was observed even in asymptomatic patients during the study period, although a large body of evidence documented that this drug is not effective in reducing the risk of COVID-19-related outcomes [17] or the time to recovery, when compared to the standard care alone [18]. It was also demonstrated that, when compared to placebo, the treatment with a single dose of azithromycin was not associated with a greater likelihood of being symptom-free within two weeks among outpatients with SARS-CoV-2 infection [19].

According to the current Italian guidelines [8], antibiotics should be used in COVID-19 patients only in the presence of proven or suspected bacterial superinfections/co-infections, which is known to occur in 8–20% of COVID-19 patients, respectively [20]. A meta-analysis of both RCTs and observational studies showed that, since around three-quarters of COVID-19 patients were treated with antibiotics, the prescribing of these drugs is significantly higher than the estimated prevalence of bacterial co-infection [21], thus posing considerable concerns about the increased risk of antibiotic resistance [22,23].

Glucocorticoids were also widely used and frequently co-prescribed with azithromycin. Evidence from the RECOVERY trial showed that the use of dexamethasone for up to 10 days in hospitalized COVID-19 patients reduced the 28-day mortality among those requiring invasive mechanical ventilation or oxygen alone, but not among those not requiring respiratory support, in which dexamethasone was associated with a trend towards an increased risk of harm [11]. Furthermore, it has been demonstrated that the risk of SARS-CoV-2 infection and COVID-19-related outcomes is higher among patients receiving high dose glucocorticoids or treated with these drugs for prolonged periods [13,14], which makes the use of glucocorticoids in early treatment of COVID-19 patients questionable. Interestingly, a phase 2, open-label RCT showed that the early administration of inhaled budesonide reduced the likelihood of needing urgent medical care and the time to recovery after early COVID-19 [24]. However, according to the EMA, evidence to establish the benefits of inhaled corticosteroids in COVID-19 patients is still sparse [25].

Around 83% of COVID-19 outpatients receiving vitamin D were incident users, i.e., they received the first vitamin D prescription after SARS-CoV-2 infection diagnosis. Cumulated evidence on the use of vitamin D supplementation as early treatment for COVID-19 outpatients is sparse and no firm conclusions can be drawn [26]. On the one side, it was suggested that low vitamin D concentrations may be associated with higher risk of SARS-CoV-2 infection and COVID-19-related outcomes [27,28]; on the other side, a recent RCT showed that, as compared with placebo, the administration of a single high dose of vitamin D3 did not significantly reduce the hospital length of stay among hospitalized COVID-19 patients [29]. As such, vitamin D supplementation in those with a documented low level may be helpful as preventive strategy, in agreement with the recommendation of the Italian Drug Agency [30], while no therapeutic effect of vitamin D in COVID-19 patients has been clearly demonstrated so far.

Some of the study drug users received the first dispensing before ID. According to a recent Italian retrospective matched-cohort study, the implementation of an early COVID-19 home-based treatment algorithm was associated with a reduced risk of hospitalization [7]. Interestingly, some of the patients receiving prescriptions of the study drugs prior to diagnosis confirmation were asymptomatic at the ID. Indeed, it may happen that relatives living with an individual testing positive for SARS-CoV-2 are preventively treated even if they are asymptomatic and even before receiving diagnostic confirmation through PCR on nasopharyngeal/throat swabs. Furthermore, in some cases, patients may be firstly diagnosed with SARS-CoV-2 infection through rapid antigen swabs and treated for COVID-19, while waiting for diagnostic confirmation through PCR on nasopharyngeal/throat swabs.

The overall cumulative rate of recovery from COVID-19 was equal to 97.9%. The breakpoint observed at 21 days from ID can be ascribed to the guidelines issued by the Italian Ministry of Health, indicating to assess patients’ recovery from SARS-CoV-2 infection through a nasopharyngeal swab at 21 days from ID and to automatically release a certificate of healing for those patients who remained asymptomatic at 21 days from ID.

The strengths of this study include the use of a large database containing real-world data of more than 40,000 COVID-19 patients from an entire province of Southern Italy during a period of more than one year, including several pandemic waves. One additional advantage of the study is the linkage of claims data with COVID-19 surveillance registry, which allowed access to accurately collect data on SARS-CoV-2 RNA testing positive patients and related outcomes. Furthermore, this is the first Italian population-based drug utilization study exploring the pharmacological management of COVID-19 outpatients over one year of the pandemic.

Nevertheless, some limitations warrant caution. Since some of the study drugs may be out-of-pocket purchases, they may not be captured by claims databases and so we may have misclassified drug use. However, the availability of data concerning the private purchase of the study drugs allowed us to quantify the effects of misclassification and confirmed that it is not possible to evaluate the use of NSAIDs (mean proportion of private purchase >80%) through claims databases, while data on the private purchase of antibiotics was in line with already published data [15]. Another limitation is that hospitalization is underestimated because data concerning hospitalizations outside Caserta LHU is updated with a mean delay of 2 years as compared to the other Caserta LHU claims databases and, for this reason we decided not to consider COVID-19 hospitalization as one of the primary outcomes.

## 5. Conclusions

In this population-based study of outpatients with confirmed SARS-CoV-2 infection from Southern Italy, we documented that the drug prescribing patterns for COVID-19 treatment in an outpatient setting from Southern Italy was not supported from current evidence on beneficial therapies for early treatment of COVID-19. Specifically, azithromycin and, to a slightly lower extent, glucocorticoids, were widely used among patients testing positive for SARS-CoV-2, even if asymptomatic, in general practice, in contrast with Italian Health Ministry recommendations, thus highlighting the need to implement strategies for improving appropriate drug prescribing in general practice.

## Figures and Tables

**Figure 2:**
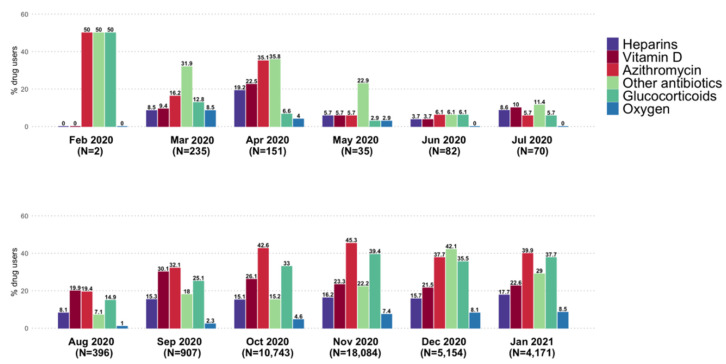
Frequency (%) of the study drug/drug class use in COVID-19 patients from Caserta Local Health Unit in the period 1 February 2020–31 January 2021, stratified by calendar month.

## Supplementary Materials

The following are available online at https://www.mdpi.com/article/10.3390/jcm11010051/s1, Table S1: List of ATC codes used to identify study drugs/drug classes, Table S2: List of ICD-9-CM, exemption codes and ATC codes used to identify comorbidities, Table S3: List of ATC codes used to evaluate prior drug use, Table S4: Demographic and clinical characteristics of patients with laboratory-confirmed SARS-CoV-2 infection in Caserta Local Health Unit during the period 21 February 2020–31 January 2021, stratified by clinical outcome, Figure S1: Cumulative COVID-19-related mortality rate within 180 days from the first laboratory-confirmed SARS-CoV-2 infection diagnosis date in Caserta Local Health Unit in the period 21 February 2020–2 April 2021, in the overall population and stratified by age groups and sex, Figure S2: Distribution of the time elapsed between the date of the first laboratory-confirmed SARS-CoV-2 infection diagnosis and the date of the first pharmacy claim, stratified by study drugs/drug classes and clinical outcomes, Figure S3: Distribution of the time elapsed between the date of the first laboratory-confirmed SARS-CoV-2 infection diagnosis and the date of the first pharmacy claim, stratified by symptoms at the SARS-CoV-2 diagnosis and study drugs/drug classes among incident drugs users, Figure S4: Distribution of the time elapsed between the date of the first laboratory-confirmed SARS-CoV-2 infection diagnosis and the date of the first pharmacy claim, stratified by symptoms at the SARS-CoV-2 diagnosis and the most commonly reported comorbidities among incident drugs users.
