# Supplementary material for: COVID-19 Patient Management in Outpatient Setting: A Population-Based Study from Southern Italy
Source: J Clin Med. 2021 Dec 23;11(1):51. doi: 10.3390/jcm11010051 (PMC8745524; doi:10.3390/jcm11010051)
Supplement: Supplementary file 1 [file jcm-11-00051-s001.zip › jcm-1522003-supplementary.pdf]

Journal of
Clinical Medicine

Supplementary Material: COVID-19 patient management in outpatient setting: a population-based study from Southern Italy

Supplementary Table S1: List of ATC codes used to identify study drugs/drug classes

Supplementary Table S2: List of ICD-9-CM, exemption codes and ATC codes used to identify comorbidities

Supplementary Table S3: List of ATC codes used to evaluate prior drug use

Supplementary Table S4: Demographic and clinical characteristics of patients with laboratory-confirmed SARS-CoV-2 infection in Caserta Local Health Unit during the period February 21, 2020 – January 31, 2021, stratified by clinical outcome

Supplementary Figure S1: Cumulative COVID-19-related mortality rate within 180 days from the first laboratory-confirmed SARS-CoV-2 infection diagnosis date in Caserta Local Health Unit in the period February 21, 2020 –April 2, 2021, in the overall population and stratified by age groups and sex

Supplementary Figure S2: Distribution of the time elapsed between the date of the first laboratory-confirmed SARS-CoV-2 infection diagnosis and the date of the first pharmacy claim, stratified by study drugs/drug classes and clinical outcomes

Supplementary Figure S3: Distribution of the time elapsed between the date of the first laboratory-confirmed SARS-CoV-2 infection diagnosis and the date of the first pharmacy claim, stratified by symptoms at the SARS-CoV-2 diagnosis and study drugs/drug classes among incident drugs users

Supplementary Figure S4: Distribution of the time elapsed between the date of the first laboratory-confirmed SARS-CoV-2 infection diagnosis and the date of the first pharmacy claim, stratified by symptoms at the SARS-CoV-2 diagnosis and the most commonly reported comorbidities among incident drugs users

Supplementary Table 1. List of ATC codes used to identify study drugs/drug classes

**Active substance/drug class**	**ATC**
Glucocorticoids	H02AB*
Heparins	B01AB*
Antibiotics	
Macrolides	J01FA*

Azithromycin	J01FA10
Tetracyclines	J01A*
Penicillins	J01C*
Other beta-lactam antibacterials	J01D*
Aminoglycosides	J01G*
Quinolones	J01M*
Combinations of antibacterials	J01RA*
Other antibacterials	J01X*
Oxygen	V03AN01
Vitamin D and analogues	A11CC*

Abbreviations: ATC = Anatomical Therapeutic Chemical classification system

Supplementary Table 2. List of ICD-9-CM, exemption codes and ATC codes used to identify comorbidities

Comorbidity	Identification		
	ICD-9 CM codes	Exemption codes	ATC codes
Pulmonary chronic diseases	480.xx-487.xx, 490.xx-491.xx, 496.xx		
Ischemic cardiopathy	410.xx., 411.xx., 412.xx., 413.xx., 414.xx	-	-
Atrial fibrillation	427.3.xx., 99.61	-	-
Heart failure	428.xx	-	-
Hypertension	401.xx., 402.xx., 403.xx., 404.xx	-	C02,* C03*, C07*, C08*, C09*
Cerebrovascular diseases	430.xx - 438.xx	-	-
Chronic kidney diseases	585.xx	023	
Diabetes	250.xx	013	A10*
Neoplasms	140.xx-209.xx	048	-
Hepatopathies	571.x-573.xx	-	-

Abbreviations: ICD-9-CM = International Classification of Diseases - 9th revision - Clinical Modification; ATC = Anatomical Therapeutic Chemical classification system

Supplementary Table 3. List of ATC codes used to evaluate prior drug use

Drugs	ATC codes
Drugs for acid-related disorders	A02*
Lipid-lowering drugs	C10*
Anticoagulants	B01AA*<br>B01AB*<br>B01AE*<br>B01AF*<br>B01AX*
Anti-platelet agents	B01AC*
Class I and III antiarrhythmics	C01B*
Antibiotics	J01*
Anti HIV drugs	J05AE*<br>J05AF*<br>J05AG*<br>J05AR*
Anti-Parkinson drugs	N04*
Antiepileptics	N03*
Antipsychotics	N05A*
Antidepressants	N06A*
Acetylsalicylic acid	B01AC06

Abbreviations: ATC = Anatomical Therapeutic Chemical classification system

Supplementary Table 4. Demographic and clinical characteristics of patients with laboratory-confirmed SARS-CoV-2 infection in Caserta Local Health Unit during the period February 21, 2020 – January 31, 2021, stratified by clinical outcome

	Overall N= 40,030 (%)	Recovered N= 39,179 (%)	Deceased N= 720 (%)	Not yet recovered N= 131 (%)
**Sex**				
Males	19,912 (50.2)	19,434 (49.6)	417 (57.9)	61 (46.6)
Females	20,118 (49.8)	19,745 (50.4)	303 (42.1)	70 (53.4)
**Median age (IQR) (years)**	44 (27-58)	43 (27-57)	78 (70-85)	53 (37-71)
**Age groups (years)**				
<18	4,663 (11.6)	4,654 (11.9)	0 (0.0)	9 (6.9)
18-44	16,137 (40.3)	16,087 (41.1)	12 (1.7)	38 (29.0)
45-64	13,032 (32.6)	12,880 (32.9)	109 (15.1)	43 (32.8)
65-80	4,541 (11.3)	4,227 (10.8)	288 (40.0)	26 (19.8)
>80	1,657 (4.2)	1,331 (3.3)	311 (43.2)	15 (11.5)
**Hospitalized patients**	745 (1.9)	534 (1.4)	205 (28.5)	6 (4.6)
**Symptoms**				
Asymptomatic	20,528 (51.3)	20,188 (51.5)	267 (37.1)	73 (55.7)
Mild	11,851 (29.6)	11,700 (29.9)	115 (16.0)	36 (27.5)
Moderate	4,981 (12.4)	4,838 (12.3)	134 (18.6)	9 (6.9)

Serious	695 (1.7)	640 (1.6)	52 (7.2)	3 (2.3)
Not available	1,975 (4.9)	1,813 (4.6)	152 (21.1)	10 (7.6)
**Medications taken**				
At least 1 antibiotic*	20,777 (51.9)	20,224 (51.6)	488 (67.8)	65 (49.6)
Azithromycin	16,843 (42.1)	16,497 (42.1)	300 (41.7)	46 (35.1)
Other antibiotics**	8,360 (20.9)	7,937 (20.3)	385 (53.5)	38 (29.0)
≥2 antibiotics	5,287 (13.2)	4,997 (12.8)	261 (36.2)	29 (22.1)
Glucocorticoids	14,412 (36.0)	14,009 (35.8)	354 (49.2)	49 (37.4)
Heparins	6,322 (15.8)	5,969 (15.2)	316 (43.9)	37 (28.2)
Vitamin D	9,486 (23.7)	9,290 (23.7)	172 (23.9)	24 (18.3)
Oxygen	2,664 (6.7)	2,298 (5.9)	347 (48.2)	19 (14.5)
Azithromycin + glucocorticoids	11,675 (29.2)	11,402 (29.1)	242 (33.6)	31 (23.7)
Azithromycin + glucocorticoids + heparins	3,848 (9.6)	3,658 (9.3)	171 (23.8)	19 (14.5)

Abbreviations: SD = standard deviation

* ATC: J01* azithromycin excluded

** ≥2 different ATCs belonging to the ATC code J01*, with a dispensing date following the date of SARS-CoV-2 infection diagnosis or 10 days earlier

Supplementary Figure 1. Cumulative COVID-19-related mortality rate within 180 days from the first laboratory-confirmed SARS-CoV-2 infection diagnosis date in Caserta Local Health Unit in the period February 21, 2020 –April 2, 2021, in the overall population and stratified by age groups and sex

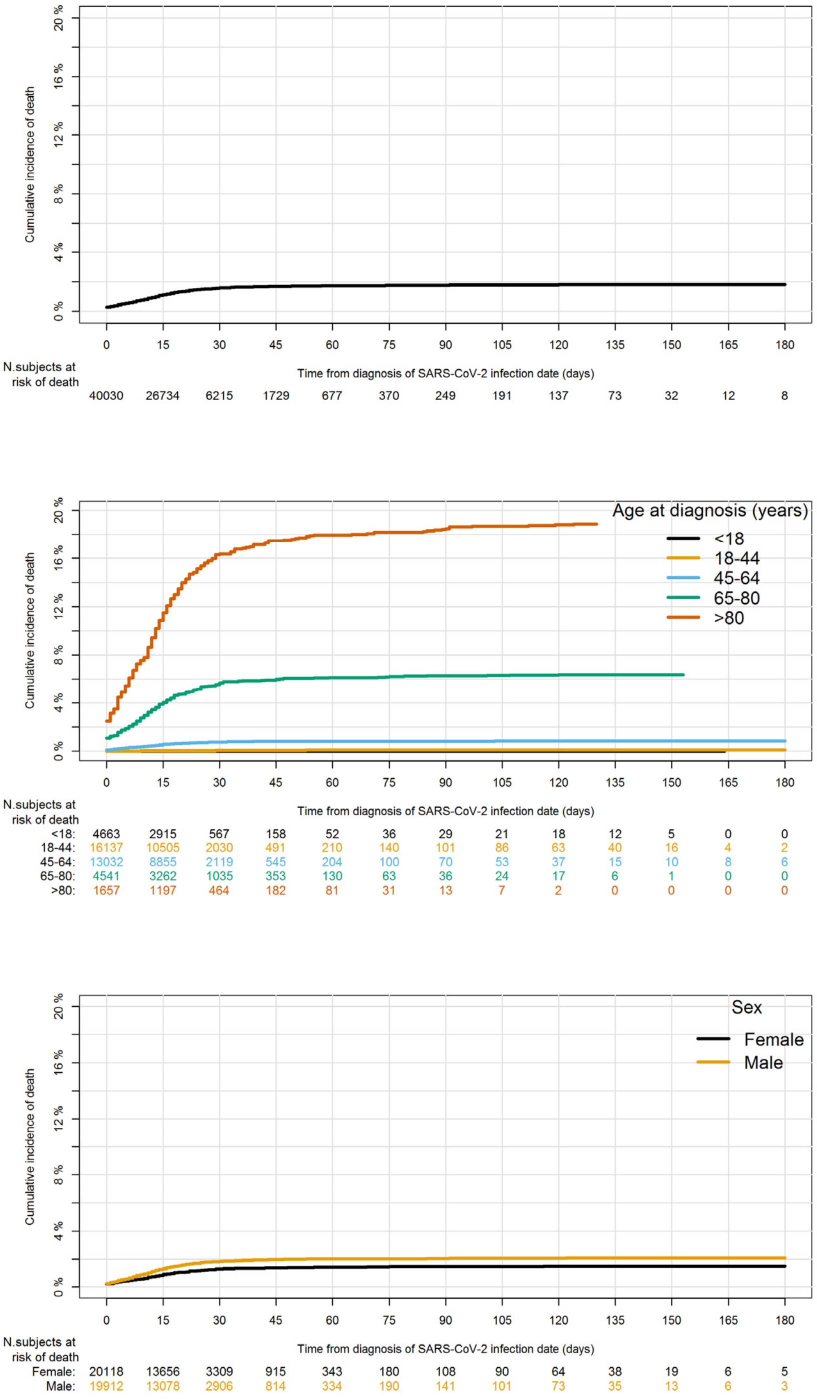

Supplementary Figure 2. Distribution of the time elapsed between the date of the first laboratory-confirmed SARS-CoV-2 infection diagnosis and the date of the first pharmacy claim, stratified by study drugs/drug classes and clinical outcomes

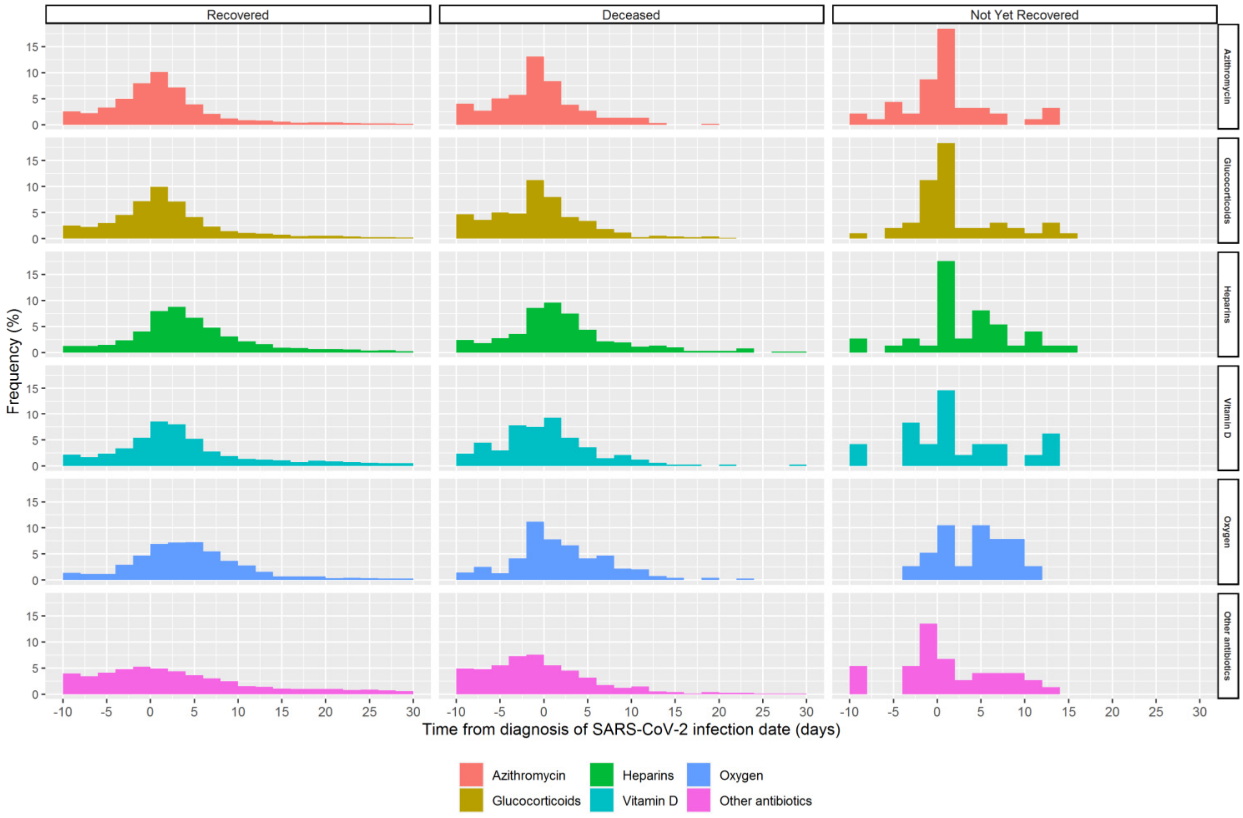

Supplementary Figure 3. Distribution of the time elapsed between the date of the first laboratory-confirmed SARS-CoV-2 infection diagnosis and the date of the first pharmacy claim, stratified by symptoms at the SARS-CoV-2 diagnosis and study drugs/drug classes among incident drugs users.

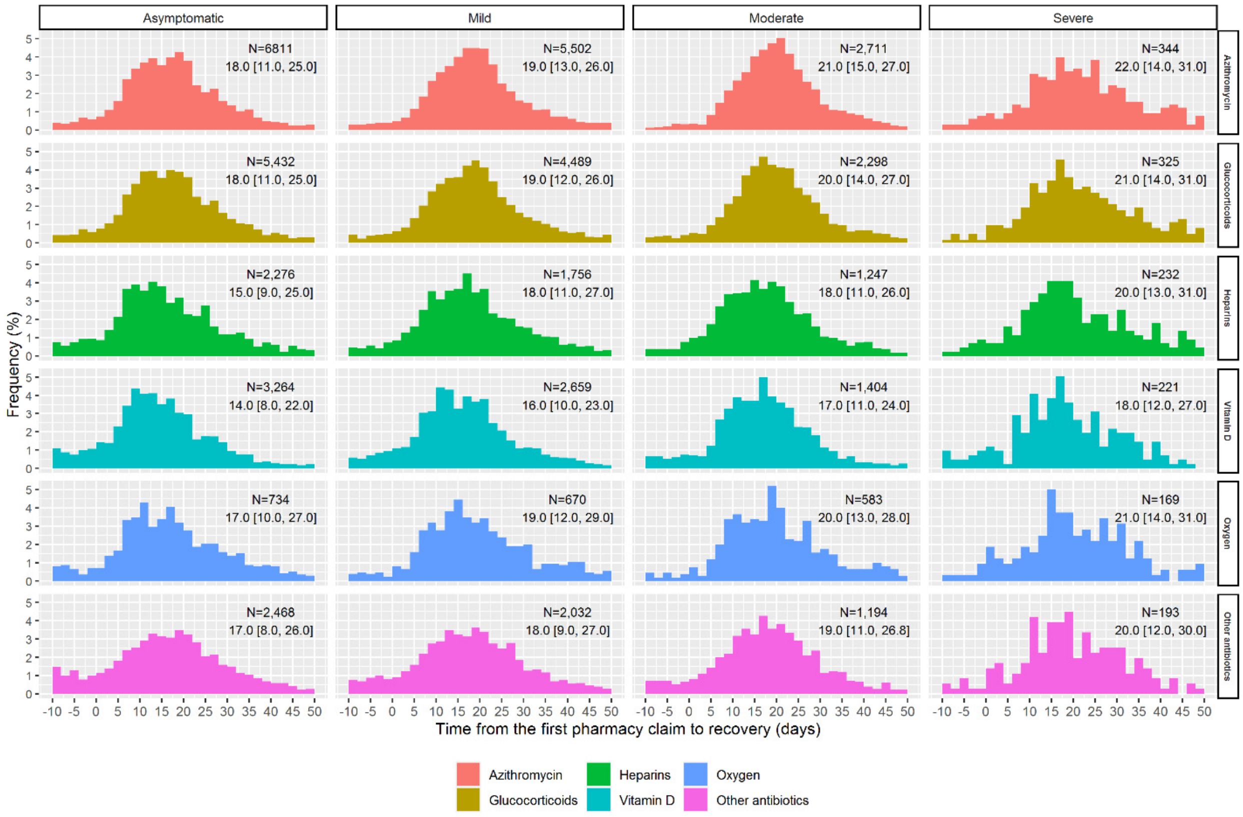

Supplementary Figure 4. Distribution of the time elapsed between the date of the first laboratory-confirmed SARS-CoV-2 infection diagnosis and the date of the first pharmacy claim, stratified by symptoms at the SARS-CoV-2 diagnosis and the most commonly reported comorbidities among incident drugs users.

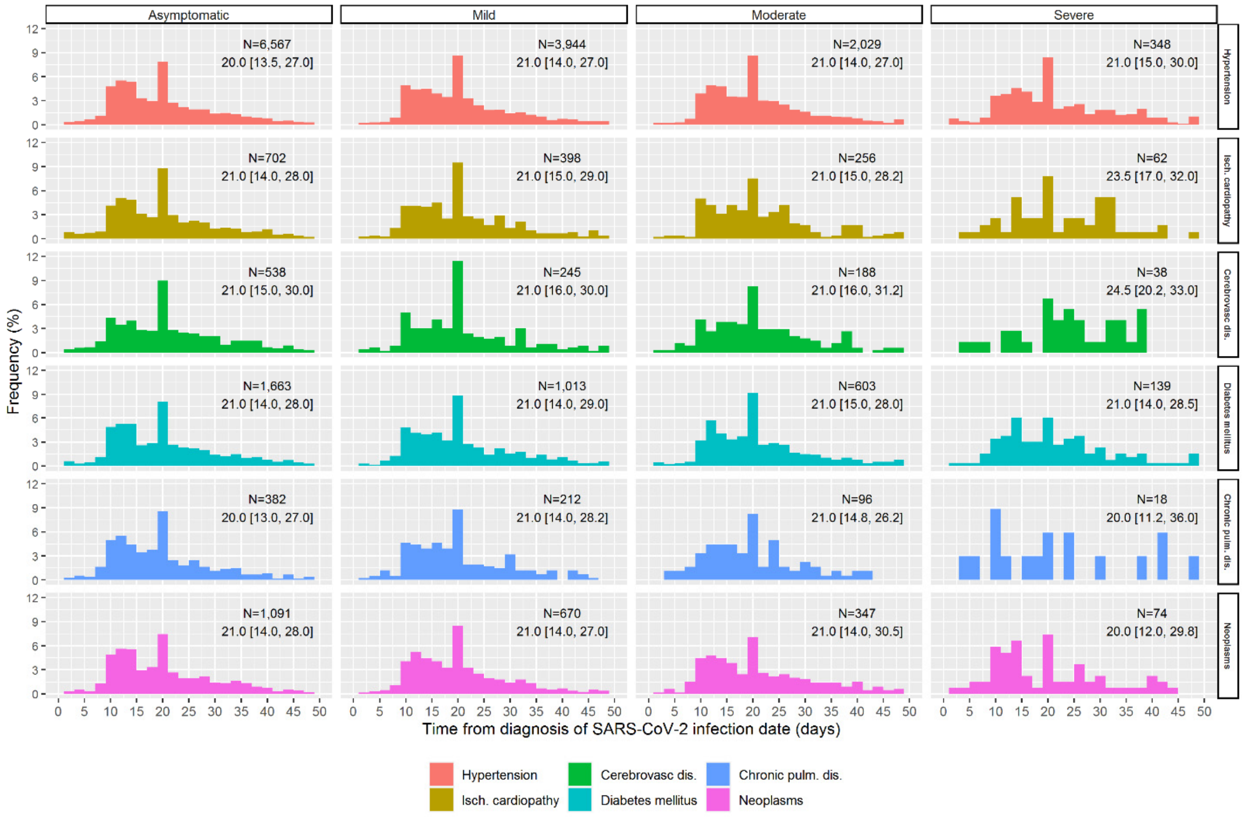

The number of users (N) and the median (along with first-third quartiles) of elapsed days are reported within each plot, respectively.
